# Bilateral Endoprosthetic Replacements of the Proximal Femur

**DOI:** 10.1080/13577149878163

**Published:** 1998-03

**Authors:** Athanasios F. Foukas, Robert J. Grimer

**Affiliations:** Royal Orthopaedic Hospital Oncology Service Royal Orthopaedic Hospital Bristol Road South Northfield, Birmingham B31 2AP UK

## Abstract

*Patient.* We report the case of a 20-year-old caucasian male with an Ewing's sarcoma in the left upper femur. After induction chemotherapy, he underwent resection of the left upper femur and insertion of a cemented proximal femoral replacement. Four years later, he presented with a solitary bone metastasis in the right upper femur. He underwent further chemotherapy followed by resection and endoprosthetic replacement of the other proximal femur. He remains disease free with excellent function almost a year after the second operation.

*Discussion.* We believe this is the first reported case of bilateral proximal femoral endoprostheses.


*Sarcoma* (1998) **2**, 49–51

CASE REPORT

### Bilateral endoprosthetic replacements of the proximal femur

ATHANASIOS F. FOUKAS & ROBERT J. GRIMER

*Royal Orthopaedic Hospital Oncology Service, Birmingham, UK*

**Abstract**

*Patient.* We report the case of a 20-year-old caucasian male with an Ewing's sarcoma in the left upper femur. After induction chemotherapy, he underwent resection of the left upper femur and insertion of a cemented proximal femoral replacement. Four years later, he presented with a solitary bone metastasis in the right upper femur. He underwent further chemotherapy followed by resection and endoprosthetic replacement of the other proximal femur. He remains disease free with excellent function almost a year after the second operation.

*Discussion.* We believe this is the first reported case of bilateral proximal femoral endoprostheses.

**Key words**: *limb-salvage surgery, endoprosthetic replacement, proximal femur.*

### Introduction

Limb-salvage surgery is now possible in 85% of patients with primary sarcomas of bone. Refinements in surgical techniques and chemotherapy regimes also mean that patients are living longer and hence the effectiveness of the limb-salvage surgery and its durability is also becoming increasingly important.[1–5] As patients survive longer, unusual patterns of metastatic disease are appearing and these present their own problems in treatment—both surgically and oncologically. We report a case of Ewing's sarcoma in which a solitary bone metastasis developed in the contra-lateral proximal femur $4\frac{1}{2}$ years after endoprosthetic replacement and chemotherapy for Ewing's sarcoma of the left proximal femur. It was treated with a second proximal femoral endoprosthesis. We believe this is the first reported case of bilateral proximal femoral endoprosthetic replacements for primary bone tumour.

### Case history

A 20-year-old white Caucasian male was referred to us in July 1991 with a 25-week history of pain in the left upper femur. Radiographic imaging and biopsy confirmed Ewing's sarcoma with extensive soft tissue disease (Figs 1 and 2). He received induction chemotherapy with IVAD (ifosfamide, vincristine, adriamycin, actinomycin D) and after four cycles he underwent resection of the proximal femur and insertion of a cemented proximal femoral replacement replacing 61% of the femur. Pathological evaluation showed greater than 95% necrosis of the tumour with wide margins of resection.

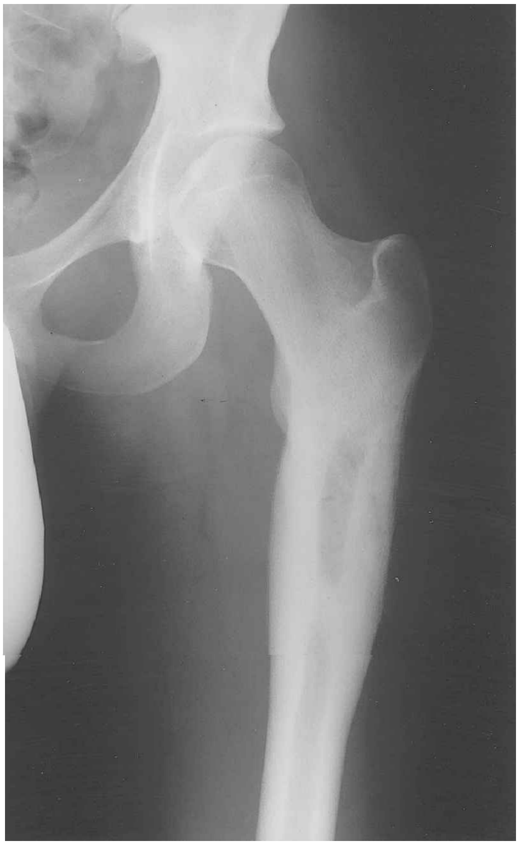

**Fig. 1.** *Initial X-rays of the primary tumour in the left femur.*

Correspondence to: A. F. Foukas, Royal Orthopaedic Hospital Oncology Service, Royal Orthopaedic Hospital, Bristol Road South, Northfield, Birmingham B31 2AP, UK. Tel: +44 121 685 4150; Fax: +44 121 685 4146.

1357-714X/98/040049-03 ©1998 Carfax Publishing Ltd

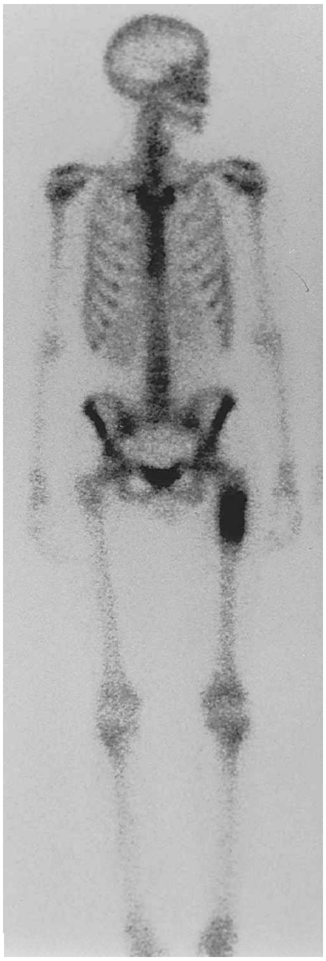

**Fig. 2.** *Bone scan: primary Ewing's sarcoma in the left upper femur.*

He did very well, regaining near normal function and achieving an Enneking functional score of 29 out of 30 (96%) at follow-up 4 years later. In February 1996, he started to get right thigh pain and was found to have a solitary bone metastasis in the right upper femur. He was started on induction chemotherapy with EVAIA (etoposide, vincristine, adriamycin ifosfamide, actinomycin D) and was kept non-weight-bearing on crutches. After the first cycle of chemotherapy, he fell and sustained a pathological fracture through the right proximal femur (Fig. 3). He was admitted to hospital for traction and further chemotherapy. After two further cycles of chemotherapy, he underwent resection of the upper femur and insertion of a second endoprosthetic replacement, this time replacing 45% of the bone. Ten days post-operatively, he was up walking on crutches and was discharged after 14 days. Histology showed 50% necrosis of the tumour with clear margins of resection. By 8 months following the second surgery the patient was walking without any sticks. Trendelenburgh is negative on the left but delayed positive on the right side. The functional score is 76% on the right and 90% on the left side (Fig. 4). After 18 months he relapsed with further widespread bony metastases.

### Discussion

The outcome of Ewings sarcoma treatment has improved dramatically with chemotherapy and surgery for patients who present free of metastatic disease. Disease-free survival of 65% at 5 years is reported.[2,6] Most relapses occur within the first 2 years.[1–3,5–7] Relapse at $4\frac{1}{2}$ years with a solitary bone metastasis is unusual but not unique. A late relapse at the same site as the initial tumour is exceedingly unusual.

The prognosis for patients who relapse is poor with 20% survival at 3 years in the CESS 86 Study.[6] However, it has been shown in the same series that if the time to relapse is greater than 24 months from original diagnosis, then the prognosis improves considerably, and in the CESS 86 series $\frac{3}{4}$ patients with bone metastasis appearing later than 24 months from diagnosis were still disease free at 4 years following relapse. Aggressive treatment with further chemotherapy and wide excisional surgery is clearly justified in these cases.

Endoprosthetic replacement of the proximal femur is a well established surgical technique for replacing tumours of the upper femur.[8–10] One of the main disadvantages of this method is the loss of muscle and in particular the removal of the abductor

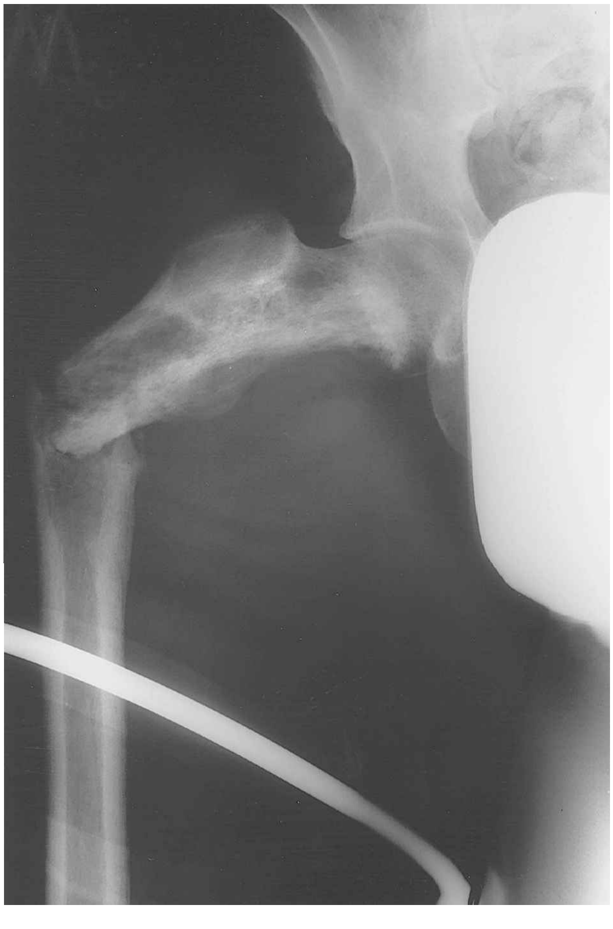

**Fig. 3.** *Pathological fracture through the secondary lesion in the right femur.*

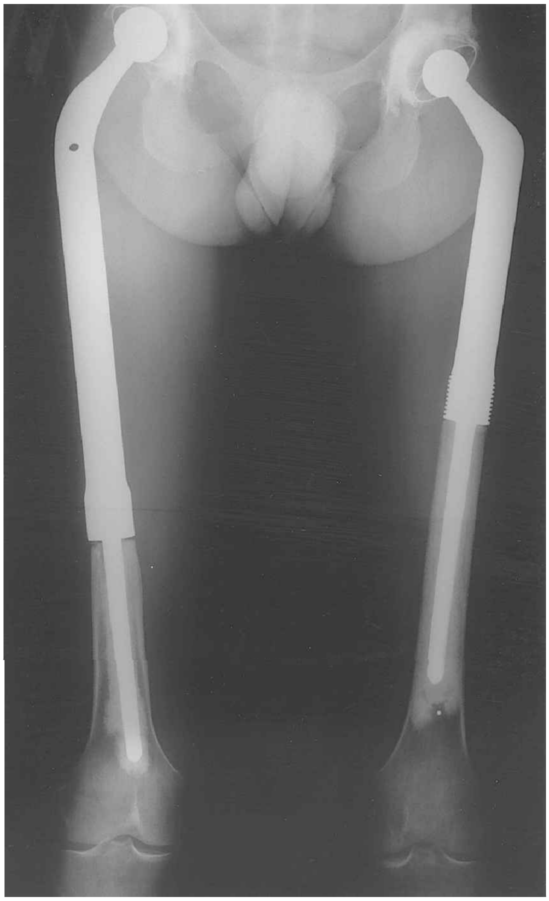

**Fig. 4.** *PA film 14 days after the right proximal femoral endoprosthesis (patient upright).*

lever arm from its insertion of the greater trochanter. In all our patients the abductors are reattached to the fascia lata and while this can achieve a good functional abductor arm particularly in younger patients, most people will have a slight Trendelenberg dip and older patients frequently need to use a walking stick.

Another approach would be to treat with chemotherapy and local radiotherapy. Damron *et al.* reported a 79% rate of pathological fracture following treatment of Ewings sarcoma of the proximal femur with radiotherapy. Increasing evidence suggests that the surgical management of primary Ewing's sarcoma does give improved rate of both survival and local control.[11,12]

We know of no case where bilateral endoprosthetic replacements of the upper femur for primary bone tumours have been carried out. The patient is young and well motivated and we would anticipate good functional recovery. The prognosis is clearly uncertain. We firmly believe that aggressive surgical reconstruction combined with chemotherapy is justified for these unusual cases.

**Acknowledgement**

Special thanks to Michaela Fischler (Student of Medicine in University of Tubingen in Germany) for the translation of the German articles into English.

**References**

1 Mirra J. *Bone tumours*. Philadelphia: Lea & Febiger, 1979:1088–117.
2 Bacci G, Ferrari S, Rosito P, Avella M. Ewing's sarcoma of the bone. Anatomoclinical study of 424 cases. *Minerva-Pediatr* 1992; 44:345–59.
3 Dunst J, Sauer R, Burgers JMV, *et al.* Radiation therapy as local treatment in Ewings sarcoma. Results of the Cooperative Ewings Sarcoma Studies CESS 81 and CESS 86. *Cancer* 1993; 67:2818–25.
4 Jurgens H, Exner U, Gadner H, *et al.* Multidisciplinary treatment of Ewings sarcoma of bone. A 6-year experience of a European Cooperative Trial. *Cancer* 1993; 61:23–32.
5 Jurgens H, Bier V, Dunst J, *et al.* The G.P.O. Cooperative Ewing's Sarcoma Studies CESS (81–86). Report after $6\frac{1}{2}$ years. *Klin Pediatr* 1988; 200:243–52.
6 Paulussen M, Braun-Munzinger G, Burdach S, *et al.* Results of treatment of primary exclusively pulmonary metastatic Ewing's sarcoma. A retrospective analysis of 41 patients. *Klin Pediatr* 1993; 205:210–6.
7 Sandoval C, Meyer WH, Parham DM, *et al.* Outcome in 43 children presenting with metastatic Ewing's sarcoma. The St Jude Children's Research Hospital experience 1962–1992. *Med Ped Oncol* 1996; 26:180–5.
8 Maurer KP, Refior HJ. Alloplastic replacement of proximal femur, indications, results and experiences. *Z Orth Grenzg* 1996; 134:21–8.
9 Unwin PS, Cannon SR, Grimer RJ, *et al.* Aseptic loosening in cemented custom made prosthetic replacements for bone tumours of lower limbs. *JBJS* 1996; 78B:5–13.
10 Chan D, Carter SR, Grimer RJ, Sneath RS. Endoprosthetic replacement for bony metastases. *Ann R Coll Surg Eng* 1992; 74:13–8.
11 Craft AW, Cotterill SJ, Bullimore JA, *et al.* Longterm results from the first UKCCSG Ewing's tumour study (ET-1). *Eur J Cancer (England)* 1997; 33:1061–9.
12 Ozaki T, Hillman A, Hoffman C, *et al.* Ewing's sarcoma of the femur. *Acta Othop Scand (Norway)* 1997; 68:20–4.

## References

[PDF_00132] Bacci G., Ferrari S., Rosito P., Avella M., Barbieri E., Picci P., Battistini A., Brach del Prever A. (1992). Sarcoma di Ewing dell'osso. Studio anatomoclinico di 424 casi.. Minerva Pediatr.

[PDF_00165] Chan D., Carter S. R., Grimer R. J., Sneath R. S. (1992). Endoprosthetic replacement for bony metastases.. Ann R Coll Surg Engl.

[PDF_00168] Craft A. W., Cotterill S. J., Bullimore J. A., Pearson D. (1997). Long-term results from the first UKCCSG Ewing's Tumour Study (ET-1). United Kingdom Children's Cancer Study Group (UKCCSG) and the Medical Research Council Bone Sarcoma Working Party.. Eur J Cancer.

[PDF_00135] Dunst J., Sauer R., Burgers J. M., Hawliczek R., Kürten R., Winkelmann W., Salzer-Kuntschik M., Müschenich M., Jürgens H. (1991). Radiation therapy as local treatment in Ewing's sarcoma. Results of the Cooperative Ewing's Sarcoma Studies CESS 81 and CESS 86.. Cancer.

[PDF_00143] Jürgens H., Bier V., Dunst J., Harms D., Jobke A., Kotz R., Kühl J., Müller-Weihrich S., Ritter J., Salzer-Kuntschik M. (1988). Die GPO cooperativen Ewing-Sarkom Studien CESS 81/86: Bericht nach 6 1/2 Jahren.. Klin Padiatr.

[PDF_00139] Jürgens H., Exner U., Gadner H., Harms D., Michaelis J., Sauer R., Treuner J., Voûte T., Winkelmann W., Winkler K. (1988). Multidisciplinary treatment of primary Ewing's sarcoma of bone. A 6-year experience of a European Cooperative Trial.. Cancer.

[PDF_00149] Paulussen M., Braun-Munzinger G., Burdach S., Deneke S., Dunst J., Fellinger E., Göbel U., Mittler U., Treuner J., Voûte P. A. (1993). Behandlungsergebnisse beim primär ausschliesslich pulmonal metastasierten Ewingsarkom. Eine retrospektive Analyse von 41 Patienten.. Klin Padiatr.

[PDF_00153] Sandoval C., Meyer W. H., Parham D. M., Kun L. E., Hustu H. O., Luo X., Pratt C. B. (1996). Outcome in 43 children presenting with metastatic Ewing sarcoma: the St. Jude Children's Research Hospital experience, 1962 to 1992.. Med Pediatr Oncol.

